# Discovery of Novel Bovine Viral Diarrhea Inhibitors Using Structure-Based Virtual Screening on the Envelope Protein E2

**DOI:** 10.3389/fchem.2018.00079

**Published:** 2018-03-26

**Authors:** Mariela Bollini, Emilse S. Leal, Natalia S. Adler, María G. Aucar, Gabriela A. Fernández, María J. Pascual, Fernando Merwaiss, Diego E. Alvarez, Claudio N. Cavasotto

**Affiliations:** ^1^Laboratorio de Química Medicinal, Centro de Investigaciones en Bionanociencias, Consejo Nacional de Investigaciones Científicas y Técnicas, Ciudad de Buenos Aires, Argentina; ^2^Laboratory of Computational Chemistry and Drug Design, Instituto de Investigación en Biomedicina de Buenos Aires, Consejo Nacional de Investigaciones Científicas y Técnicas, Partner Institute of the Max Planck Society, Ciudad de Buenos Aires, Argentina; ^3^Instituto de Investigaciones Biotecnológicas, Universidad Nacional de San Martín, Consejo Nacional de Investigaciones Científicas y Técnicas, San Martín, Argentina

**Keywords:** BVDV entry inhibitors, structure-based virtual screening, molecular dynamics simulation, envelope protein, molecular docking

## Abstract

Bovine viral diarrhea virus (BVDV) is a member of the genus Pestivirus within the family Flaviviridae. BVDV causes both acute and persistent infections in cattle, leading to substantial financial losses to the livestock industry each year. The global prevalence of persistent BVDV infection and the lack of a highly effective antiviral therapy have spurred intensive efforts to discover and develop novel anti-BVDV therapies in the pharmaceutical industry. Antiviral targeting of virus envelope proteins is an effective strategy for therapeutic intervention of viral infections. We performed prospective small-molecule high-throughput docking to identify molecules that likely bind to the region delimited by domains I and II of the envelope protein E2 of BVDV. Several structurally different compounds were purchased or synthesized, and assayed for antiviral activity against BVDV. Five of the selected compounds were active displaying IC_50_ values in the low- to mid-micromolar range. For these compounds, their possible binding determinants were characterized by molecular dynamics simulations. A common pattern of interactions between active molecules and aminoacid residues in the binding site in E2 was observed. These findings could offer a better understanding of the interaction of BVDV E2 with these inhibitors, as well as benefit the discovery of novel and more potent BVDV antivirals.

## Introduction

Bovine viral diarrhea virus (BVDV) is a worldwide distributed pathogen of cattle. Together with classical swine fever virus (CSFV) and border disease virus (BDV) of sheep, BVDV belongs to the genus Pestivirus of the Flaviviridae family. The pestiviral genome is a positive, single-stranded RNA molecule of about 12.3 kb in length encoding a single polyprotein that is processed into individual viral proteins: Npro -C-Erns -E1-E2-p7-NS2-NS3-NS4A-NS4B-NS5A-NS5B (Collett et al., 1988). Pestivirus particles consist of a lipid bilayer with envelope glycoproteins Erns, E1, and E2 surrounding the nucleocapsid, composed by the capsid protein C and the RNA genome (Callens et al., 2016). BVDV infection is distributed worldwide resulting in major economic losses to the livestock industry. The virus is primarily a pathogen of cattle and the clinical manifestations are presented as acute infection, fetal infection, or mucosal disease (Lanyon et al., 2014). Based on genetic and antigenic differences, BVDV is segregated into genotypes 1 and 2. For each of these genotypes, cytopathic and non-cytopathic biotypes are distinguished according to the capacity of virus infection to induce cell death in culture (Ridpath, 2003). Non cytopathic (ncp) BVDV biotypes cause acute infections in adult animals and can be transmitted across the placenta to the fetus. Fetal infection is particularly relevant and it can lead to congenital malformations and abortion, or to the birth of persistently infected (PI) calves that spread and maintain the disease in cattle populations (Lanyon et al., 2014). Cytopathic (cp) BVDV biotypes arise in PI cattle from recombination events in the infecting ncpBVDV genome, and are associated with the development of fatal mucosal disease (Becher and Tautz, 2011).

Control and prevention of BVDV infection should combine systematic vaccination with detection and culling of persistently infected cattle from herds (Newcomer and Givens, 2013). However, immunization is complicated due to the wide antigenic diversity of the virus, and fails to target the emergence of persistently infected animals (Fulton et al., 2003; Newcomer et al., 2017). Previous studies showed that antivirals directed against the pestivirus polymerase NS5B provide immediate protection from viral challenge (Newcomer et al., 2012), thus prophylactic treatment with antivirals represents an alternative for therapeutic intervention in outbreaks of BVDV.

Computer-aided drug design has become an integral part of drug discovery and development in the pharmaceutical and biotechnology industry, and is nowadays extensively used in lead identification and optimization (Cavasotto and Orry, 2007; Jorgensen, 2009; Spyrakis and Cavasotto, 2015). Virus envelope proteins are attractive targets for the development of antiviral agents, and structure-based drug design has been successfully used to identify small molecule ligands of envelope proteins that block entry of flaviviruses (Zhou et al., 2008; Kampmann et al., 2009; Leal et al., 2017). With the aim of finding novel targets for pestivirus drug design, we focused on the *in silico* identification of antivirals directed against the envelope protein E2 of BVDV. E2 mediates receptor recognition on the cell surface and is required for fusion of virus and cell membranes after the endocytic uptake of the virus during entry (Ronecker et al., 2008; Wang et al., 2009). In this work, we expand on a structure-based approach to seek hit small-molecules that dock into the druggable pocket at the interface between domains I and II of the envelope protein E2 of BVDV (Pascual et al., 2018). Around a million compounds from different chemical libraries were screened in a high-throughput docking (HTD) fashion. This led to the selection of nineteen lead candidates that were either purchased or synthesized, and evaluated in a reporter-based assay for antiviral activity. The likely interaction of active compounds with the protein E2 was further characterized by molecular dynamics (MD) simulations. The approach presented here led to the identification of five of novel compounds with anti-BVDV activity displaying IC_50_ values in the low to mid-micromolar range.

## Materials and methods

### Computational chemistry

#### Molecular system preparation

All simulations were based on the crystal structure of the pestivirus of the envelope glycoprotein E2 from BVDV (PDB 2YQ2) (El Omari et al., 2013). Protein domains were designated from the N- to the C-terminus of E2 as I, II and III according to the nomenclature used by Li et al. (2013). The molecular system was described in terms of torsional coordinates using the ECEPP/3 force field (Nemethy et al., 1992) as implemented in the ICM program (version 3.7-2c, MolSoft LLC, La Jolla, CA; Abagyan et al., 1994), and prepared in a similar fashion as earlier works (He et al., 2012; Brand et al., 2013; Leal et al., 2017; Pascual et al., 2018). Hydrogen atoms were added to the receptor structure followed by local energy minimization. All Asp and Glu residues were assigned a −1 charge, and all Arg and Lys residues were assigned a +1 charge. Histidine tautomers were assigned according to the hydrogen bonding pattern.

#### High-throughput docking

As in an earlier work (Pascual et al., 2018), docking was performed within Site I located at the interface of domains I and II of E2. All water molecules and co-factors were deleted. A flexible-ligand:rigid-receptor docking methodology as implemented in ICM was used. The receptor was represented by six potential energy maps, while the docked molecule was considered flexible and subjected to global energy minimization within the field of the receptor using a Monte Carlo protocol (Abagyan et al., 1994; Cavasotto et al., 2006); thus, the intra- and inter-molecular energy of the molecule are minimized. Each molecule was assigned an empirical docking score according to its fit within the binding site (Totrov et al., 2001). Two independent runs of HTD were performed to improve convergence of the global optimization energy, while the best score per molecule was kept.

#### Small-molecule libraries and filtering

The ZINC (Irwin and Shoichet, 2005) (accessed Nov. 2014), Maybridge (http://www.maybridge.com/), and in house databases were chosen for HTD. They were first filtered to remove the compounds containing inorganic atoms, PAINS (Filtering Pan-assay interfering substances) structures, and other reactive groups. Then the complete virtual library was pre-filtered for properties based on Lipinski's rules (Lipinski et al., 1997). Finally about a total of one million small-molecules were used. The PAINS filter was implemented through the online server FAF-Drugs3 (Lagorce et al., 2015).

#### Molecular dynamics

MD simulations were performed using GROMACS v5.1 package (Abraham et al., 2015) using the Amber99SB force field (Hornak et al., 2006). The system was solvated with the SPCE water model in a triclinic box, extending 10 Å from the protein, and neutralized adding sufficient NaCl counter ions to reach 0.15 M concentration. Bond lengths were constrained using the LINCS algorithm allowing a 2 fs time-step. Long-range electrostatics interactions were taken into account using the particle-mesh Ewald (PME) approach. The non-bonded cut-off for Coulomb and Van der Waals interactions were both 10 Å, and the non-bonded pair list was updated every 25 fs. Energy minimization was conducted through the steepest-descent algorithm, until the maximum force decayed to 1,000 [kJ mol^−1^ nm^−1^]. Then an equilibration of the whole system was performed by 500 ps of NVT simulation followed by 500 ps of NPT simulation. Temperature was kept constant at 300 K using a modified Berendsen thermostat (Berendsen et al., 1984) with a coupling constant of 0.1 ps. Constant pressure of 1 bar was applied in all directions with a coupling constant of 2.0 ps and a compressibility of 4.5 10^−5^ bar^−1^.

### Biological evaluation

#### Cell culture

MDBK cells (Bos taurus kidney, ATCC CCL-22) were purchased from ATCC and grown in Dulbecso's modified Eagle medium (DMEM) supplemented with 10% fetal bovine serum and antibiotics under 5% CO2 at 37°C. For infections, cells were cultivated in DMEM supplemented with 2% Horse serum and antibiotics under 5% CO2 at 37°C.

#### Cytotoxicity assay

Cell viability assays were performed on confluent cell cultures in 96 well plates (~15,000 cells per well). For each compound, cells were treated with serial dilutions of the compound in quadruplicates and incubated at 37°C for 3 days. Then, cell viability was measured using crystal violet staining. Briefly, cells were fixed with 10% formaldehyde, stained with crystal violet solution (20% Ethanol, 0.1% Crystal Violet), and after washing, the absorbance at 595 nm was recorded for each well in a spectrophotometer. Assays were conducted at least in duplicates, and the cytotoxic concentration 50 (CC_50_) was estimated by a nonlinear regression fitting of five data points as the compound concentration necessary to reduce cell viability by 50% compared to control non-treated cells.

#### Reporter-based assay for antiviral activity

Antiviral activity was evaluated in a reporter-based assay using a recombinant virus expressing GFP, cpBVDV/Npro GFP (Pascual et al., 2018). MDBK cells were seeded onto 24 well plates, infected with cpBVDV/Npro GFP at a multiplicity of infection of 0.1 in the presence of increasing concentrations of compounds. At 48 h post-infection cells were thoroughly washed, lifted with trypsin 0.05% and fixed using 4% paraformaldehyde in PBS. The fluorescence signal was measured using a flow cytometer (CyFlow® Space, Partec) at a detection spectrum of 488 nm. Data were analyzed in the FlowJo 7.6.2 software package. The inhibitory concentrations 50 (IC50s) for the compounds tested in the assay were calculated from curves constructed by plotting the percentage of infected cells versus the concentration of compound as the compound concentration necessary to reduce the number of infected cells by 50% compared to control non-treated cells.

### Chemistry

#### General information

NMR spectra were recorded on Bruker Biospin 600 MHz AVIII600, Bruker advance II 500 MHZ and Bruker 300 MHZ spectrometers at room temperature. Chemical shifts (δ) are reported in ppm and coupling constants (J) in Hertz. Column chromatography was carried out employing Merck silica gel (Kieselgel 60, 63–200 μm). Precoated silica gel plates F-254 were used for thin-layer analytical chromatography. The mass spectrometer utilized was a Xevo G2S QTOF (Waters Corporation, Manchester, UK) with an electrospray ionization (ESI) source. The mass spectrometer was operated in positive and negative ion modes with probe capillary voltages of 2.5 and 2.3 kV, respectively. The purity (≥95%) of all final synthesized compounds was determined by reverse phase HPLC, using a Waters 2487 dual λ absorbance detector with a Waters 1,525 binary pump and a Phenomenex Luna 5 μ C18(2) 250 × 4.6 mm column. Samples were run at 1 mL/min using gradient mixtures of 5–100% of water with 0.1% trifluoroacetic acid (TFA) (A) and 10:1 acetonitrile:water with 0.1% TFA (B) for 22 min followed by 3 min at 100% B. UV spectra were measured with a Shimadzu 3600 UV/vis/NIR spectrophotometer.

#### Synthetic procedures of new compounds from our in house library

##### Synthesis of (E)-2-(4-(dimethylamino)benzylidene)-N-(4-(trifluoromethyl)phenyl)hydrazinecarbothioamide (11)

Synthesis of N-(4-trifluoromethoxyphenyl)hydrazinecarbothioamide (**19**) Sodium hydroxide (0.14 g, 3.4 mmol) and carbon disulphide (0.2 mL, 2.8 mmol) were added to a solution of 4-(trifluoromethoxy)aniline **18** (0.50 g, 2.8 mmol) in DMF (5 mL). The mixture was stirred at room temperature for 1 h. Then, hydrazine hydrate (0.5 mL, 8.5 mmol) was added and stirring continued at 70°C for 1 h. After water addition compound **19** precipitate and the solid was filtrated off. The crude was recristallized from ethanol:water (0.28 g, 39.1 %). ^1^H NMR (500 MHz, CDCl_3_) δ 9.30 (s, 1H), 7.82 (s, 1H), 7.68 (d, *J* = 7.1 Hz, 2H), 7.24 (d, *J* = 8.7 Hz, 2H), 4.01 (s, 2H). To a solution of **19** (0.10 g; 0.40 mmol) in ethanol (3 mL) was added 4-dimethylaminobenzaldehyde (0. 65 g, 0.44 mmol). The mixture was stirred under reflux for 1 h. The reaction was then cooled to room temperature, and precipitate solid was filtered and washed with cyclohexane to give **11**, which was recristallized from ethanol. (0.07 g, 43.5 %). ^1^H NMR (600 MHz, CDCl_3_) δ 9.37 (s, 1H), 9.20 (s, 1H), 7.79 (s, 1H), 7.76 (dd, *J* = 8.9, 2.1 Hz, 2H), 7.57 (dd, *J* = 8.9, 2.1 Hz, 2H), 7.27 (d, *J* = 8.5 Hz, 2H), 6.72 (dd, *J* = 8.9, 1.9 Hz, 2H), 3.07 (s, 6H). ^13^C NMR (151 MHz, CDCl_3_) δ 175.2, 152.2, 146.5, 144.2, 144.1, 136.75, 129.0, 125.4, 125.3, 121.3, 121.2, 121.2, 120.1, 119.6, 111.8, 111.7, 40.1, 40.09. HR-MS (ES) calcd for C_17_H_18_F_3_N_4_OS [M+H]^+^ 383.1153, found 383.1141.

##### 4-((5-methylisoxazol-3-yl)amino)-4-oxobutanoic acid. (14)

To a solution of isoxazol-5-amine (0.20 g, 2.0 mmol) **20** in dioxane (5 mL) was added succinic anhydride (0.20 g, 2.0 mmol). The mixture was stirred at 80–90°C overnight. The solvent was evaporated and the obtained yellowish solid was suspended in water, collected by filtration, and crystallized from ethanol to give the pure product as a white solid (0.088 g, 0.44 mmol, 22%). ^1^H NMR (600 MHz, dmso-d_6_) δ 12.14 (s, 1H), 10.87 (s, 1H), 6.58 (s, 1H), 2.55 (t, *J* = 6.5 Hz, 2H), 2.34 (s, 3H). The other methylene group was determined by HSQC, due to the overlapping with solvent signal (SI). ^13^C NMR (151 MHz, dmso-d_6_): δ 173.4, 170.3, 169.2, 158.1, 96.2, 30.4, 28.4, 12.1. HRMS (ES) *m/z* calc. for C_8_H_10_N_2_O_4_Na [M+Na]^+^: 221.0538; found: 221.0533, C_8_H_11_N_2_O_4_ [M+H]^+^: 119.0719; found: 199.0714.

##### 4-chloro-N-(isoxazol-5-yl)benzamide (15)

A mixture of *p*-chloro benzoic acid (1.0 g, 6.4 mmol), and an excess of thionyl chloride (4.92 g, 3 mL, 41.7 mmol) was refluxed for 2 h. The excess of thionyl chloride was distilled in vacuo and the acyl chloride was used without further purification. To a solution of *p*-chlorobenzoyl chloride **23** in MeCN (10 mL) was added Cs_2_CO_3_ (3.7 g, 19 mmol) and isoxazol-5-amine **20** (0.63 g, 6.4 mmol) at 0°C and the obtained suspension was stirred at r.t. overnight. Then, the reaction mixture was concentrated under vacuo and the obtained residue was treated with water and extracted with EtOAc (4 × 20 mL). The organic layers were dried over Na_2_SO_4_, filtered-off and concentrated under vacuo to give a residue that was purified by silica gel column chromatography eluting with cHex/EtOAc (95:5–70:30). The product was obtained as a white solid (0.146 g, 0.63 mmol, 10%). ^1^H NMR (600 MHz, dmso-d_6_) δ 11.39 (s, 1H), 8.02 (d, *J* = 8.6 Hz, 2H), 7.60 (d, *J* = 8.6 Hz, 2H), 6.74 (s, 1H), 2.41 (s, 3H). ^13^C NMR (151 MHz, dmso-d_6_) δ 169.4, 164.2, 158.5, 137.1, 131.9, 129.9, 128.5, 96.9, 12.1. HRMS (ES) *m/z* calc. for C_11_H_9_ClN_2_O_2_Na [M+Na]^+^: 259.0250; found: 259.0247, C_11_H_10_ClN_2_O_2_ [M+H]^+^ 237.0431; found: 237.0428.

##### N-(4-(trifluoromethoxy)phenyl)furan-2-carboxamide (16)

Thionyl chloride (0.1 mL, 1.34 mmol) was added dropwise to a mixture of 2-furoic acid **20** (0.15 g, 1.34 mmol) and triethylamine (0.26 mL, 1.82 mmol) in DCM (5 mL) under N_2_ atmosphere. The reaction mixture was stirred at room temperature for 5 h. The crude was added to another flask containing 4-(trifluoromethoxy)aniline (0.16 mL, 1.22 mmol) and triethylamine (0.34 mL, 2.43 mmol) in DCM (5 mL). The reaction mixture was stirred at room temperature overnight. After complete reaction, the solvent was the removed under reduced pressure, water added and extracted with dichloromethane. The organic layer was sequentially washed with brine, dried over anhydrous Na_2_SO_4_ and concentrated in vacuo. The crude was purified by column chromatography (SiO_2_, dichloromethane) to give a white solid **16** (0.25 g, 75.6 %). ^1^H NMR (600 MHz, DMSO-*d*_6_) δ 10.38 (s, 1H), 7.96 (d, *J* = 0.9 Hz, 1H), 7.88 (dd, *J* = 9.1, 2.0Hz, 2H), 7.40–7.32 (m, 3H), 6.72 (dd, *J* = 3.4, 1.7 Hz, 1H). ^13^C NMR (126 MHz, CDCl_3_) δ 156.0, 147.4, 145.4, 144.3, 136.0, 121.8, 121.5, 121.0, 119.4, 115.6, 112.7. HR-MS (ES) calcd for C_12_H_8_F_3_NO_3_Na [M+Na]^+^ 294.0354, found 294.0345. Synthetic procedure of compounds **5, 12, 13** and **17** from the ZINC library are described in Supporting information (Scheme S1 and S2).

**Scheme 1:**
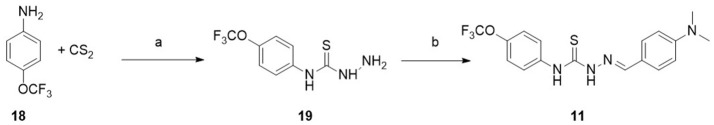
Synthesis of compound **11**. Reagents and conditions. (a) CS_2_, NaOH, DMF, 25°C, 1 h. NH_2_NH_2_, 70°C, 1 h. (b) 4-dimethylaminobenzaldehyde, EtOH, Reflux, 1 h.

**Scheme 2:**
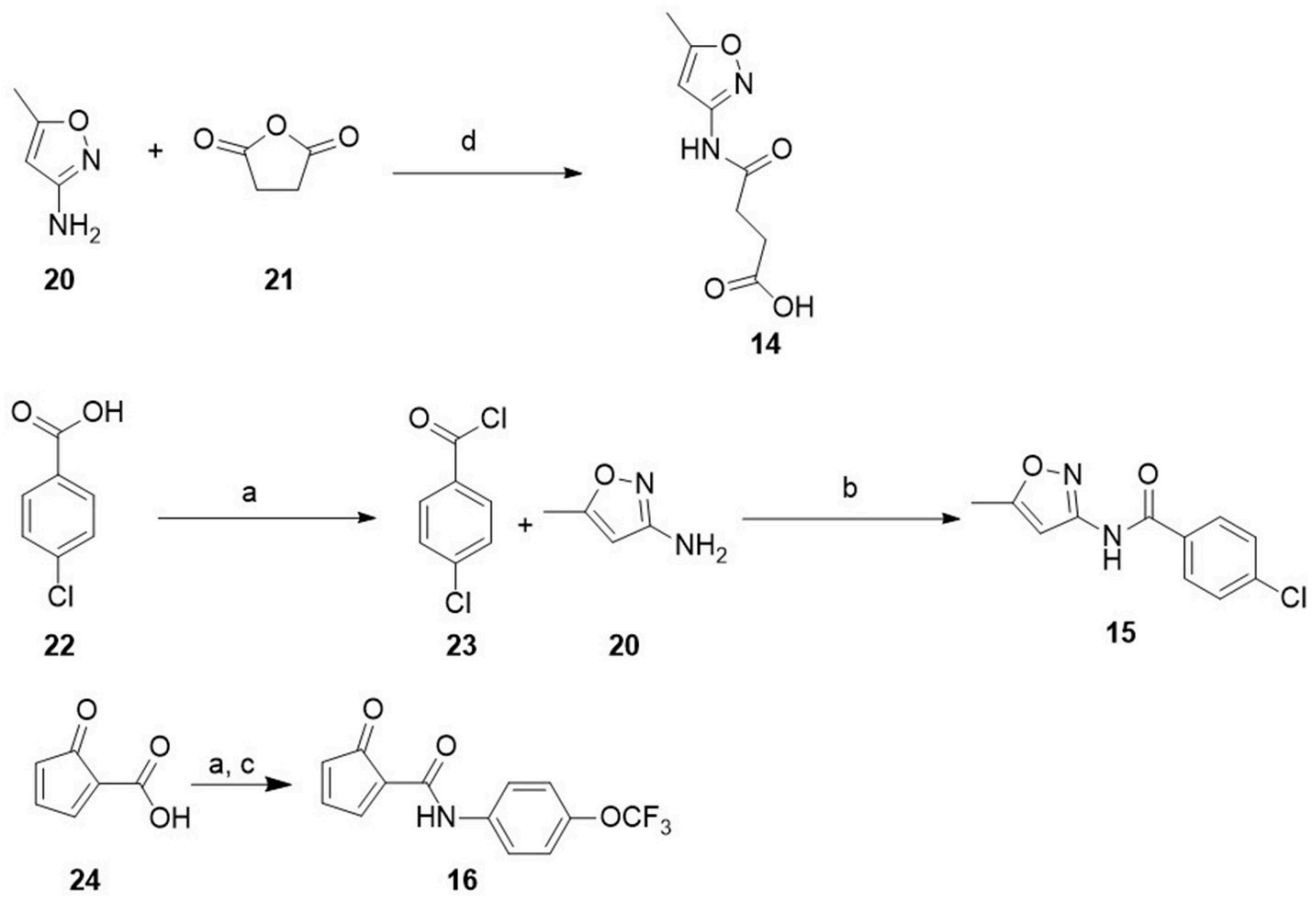
Synthesis of compound **15** and **16**. Reagents and conditions. (a) SOCl_2_, TEA, DCM, N_2_, rt, 5 h; (b) Cs_2_CO_3_, ACN, rt, overnight; (c) 4-(trifluoromethoxy)aniline, TEA, DCM, rt, overnight; (d) dioxane 80–90°C, 5 h.

## Results and discussion

### Computer-aided indentification, chemical synthesis, and biological evaluation of novel inhibitors

We employed a multistep HTD screening framework to efficiently identify novel inhibitors of the E2 protein using commercially available (ZINC and Maybridge chemical libraries) and synthetic druglike compounds (from our in house library) using the available structural data of the BVDV virus envelope protein E2. Initially, several chemical filters were applied on the chemical libraries to remove pan assay interference compounds (PAINS) (Baell and Holloway, 2010), compounds containing inorganic atoms, unwanted functionalities, reactive groups, and compounds having (i) MW <500 Dalton; (ii) more than one violation or the Lipinsky rules (Lipinski et al., 1997), or (iii) more than two violation of the rule of three, or more than six #STARS using the program QikProp (Jorgensen, 2005). The #stars parameter indicates the number of property descriptors computed by QikProp that fall outside the optimum range of values for 95% of known drugs.

**Figure 1:**
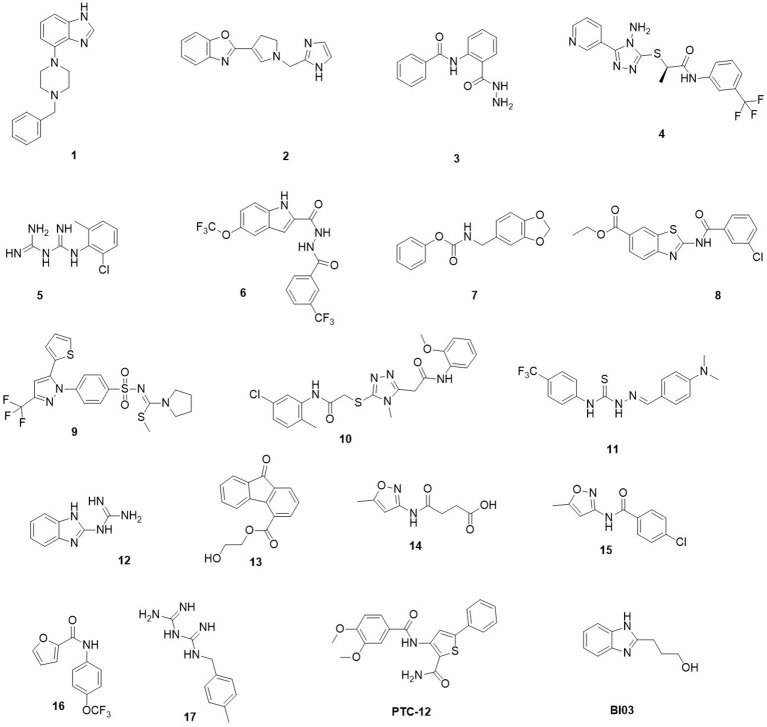
Chemical structures of the **19** hits from docking-based virtual screening.

The selected molecules were subjected to independent parallel HTD cycles and the top 1,000 scoring compounds were further analyzed. To ensure diversity, these highly ranked compounds were clustered based on chemical similarity using ICM. For each cluster, several compounds were selected manually based on commercial availability, synthetic tractability for potential modifications, interaction with binding site amino acids and adequate pharmacological characteristics for drug candidates. Finally, 19 compounds were purchased from vendors (**1**–**10**) or synthesized (**11**–**16**), and then evaluated in a reporter-based assay for antiviral activity (Figure 1, Table 1). Compounds **5** and **17** were obtained via reaction of the corresponding amine and dicyandiamide under acidic conditions to give the required phenylbiguanide (**5**, **17**) in high yields. 2-Guanidinobenzimidazole (**12**) was prepared by the cyclocondensation of o-phenylendiamine with cyanoguanidine (Scheme S1 of Supplementary Material) according to the method reported by King et al. (1948). Synthesis of new compounds is shown in Scheme 1. Compound (**11**) was obtained by ccondensation of thiosemicarbazide with 4-(dimethylamino)benzaldehyde. Compounds **14–16** were prepared by acylation of the corresponding amine with the adequate carboxilic acid chloride (**15**, **16**) or by reaction with succinic anhydride (**14**) (see Scheme 2).

**Table 1:** Antiviral activity against BVDV.

**Compound**	**IC_50_(μM)^a^ BVDV**	**CC_50_ (μM)^b^**
1	NA	>50
2	44.3 ± 10.3	>50
3	NA	>50
4	30.1 ± 2.9	>50
5	NA	>50
6	ND	7.2 ± 1.3
7	NA	>50
8	20.2 ± 3.6	>50
9	NA	>50
10	NA	>50
11	23.9 ± 7.9	>50
12	44.4 ± 11.5	>50
13	NA	61.5 ± 1.4
14	NA	>50
15	NA	>50
16	NA	49.8 ± 1.5
17	NA	>50
BI03^c^	17.6 ± 6.4	>200
PTC12^c^	0.30 ± 0.10	89.5 ± 1.1

a *IC_50_: inhibitory concentration 50%. Data represent the mean and standard deviation of at least two independent experiments*.

b *CC_50_: cytotoxic concentration 50%. Data represent the mean and standard deviation of at least two independent experiments*.

c *Pascual et al. (2018)*.

*NA indicates assayed but not active compounds; ND Not determined*.

First, we assayed selected compounds for cytotoxicity in cultured cells. Only compound **6** displayed high toxicity and was discarded from further analysis (Table 1). The remainder of the compounds were evaluated for antiviral activity in a reporter-based assay using a recombinant BVDV virus carrying GFP on its genome to infect MDBK cells (Pascual et al., 2018). Expression of GFP induced by BVDV infection was measured 2 days after infection using flow cytometry. Inhibition of BVDV infection was assessed by comparing the number of GFP positive cells in non-treated control cells and in cells treated with increasing amounts of compound. Structurally different compounds **4**, **8**, **11**, **BI03**, and **PTC12** showed activity with IC_50_ values of 30.1, 20.2, 23.9, 17.6, 0.30 μM, respectively, and no cytotoxicity was detected at 50 μM. In accordance with targeting of envelope protein function, we have previously shown that compounds BI03 and PTC12 specifically block BVDV cell entry (Pascual et al., 2018).

### Analysis of binding determinants using molecular dynamics

To further characterize the likely interaction between the new molecules and protein E2, we performed 100 ns MD simulation on the most active compounds listed on Table 1. The docked poses of the ligands within the binding site between domains I and II were used as the initial conformations. For compound **8** two conformationally different poses with very similar docking scores were used as starting conformations, and the most probable pose was assigned based on the molecular dynamics simulation results and the analysis of interactions (Liu and Kokubo, 2017). The protein and ligands remained stable in every simulation (Figure S1), displaying the ligands the following RMSF values: **PCT12**, 0.4 Å; **BI0**3, 0.4 Å; **8**, 0.3 Å; **11**, 0.2 Å. The analysis of the binding determinants of the most active compounds is described in the following paragraphs.

**Figure 2:**
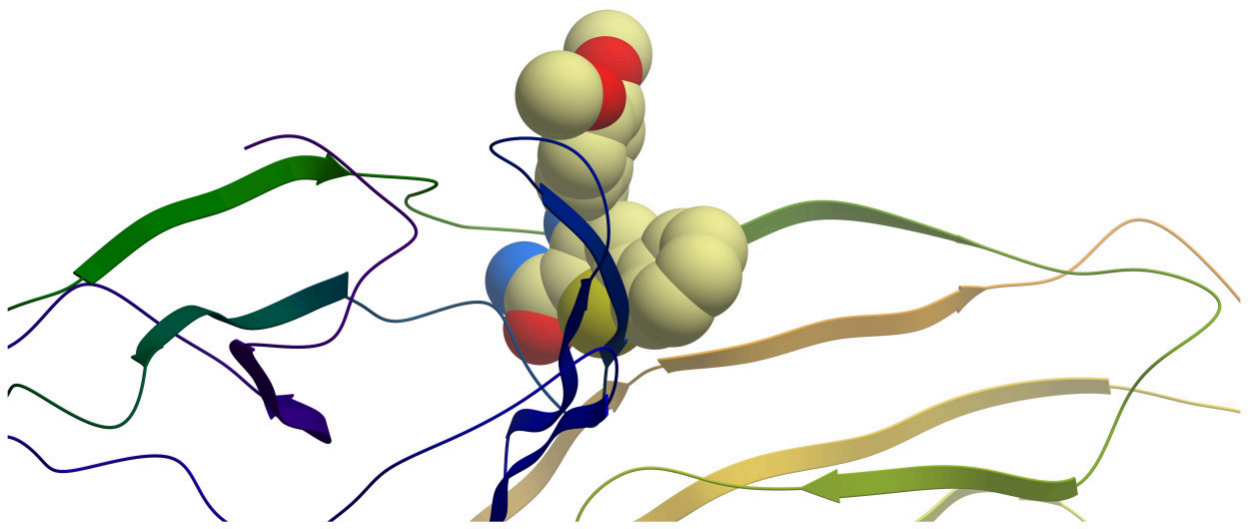
Ribbon representation of protein E2 with PTC12 ligand within the binding site. Figure prepared with ICM (MolSoft LLC, La Jolla, CA).

**Figure 3:**
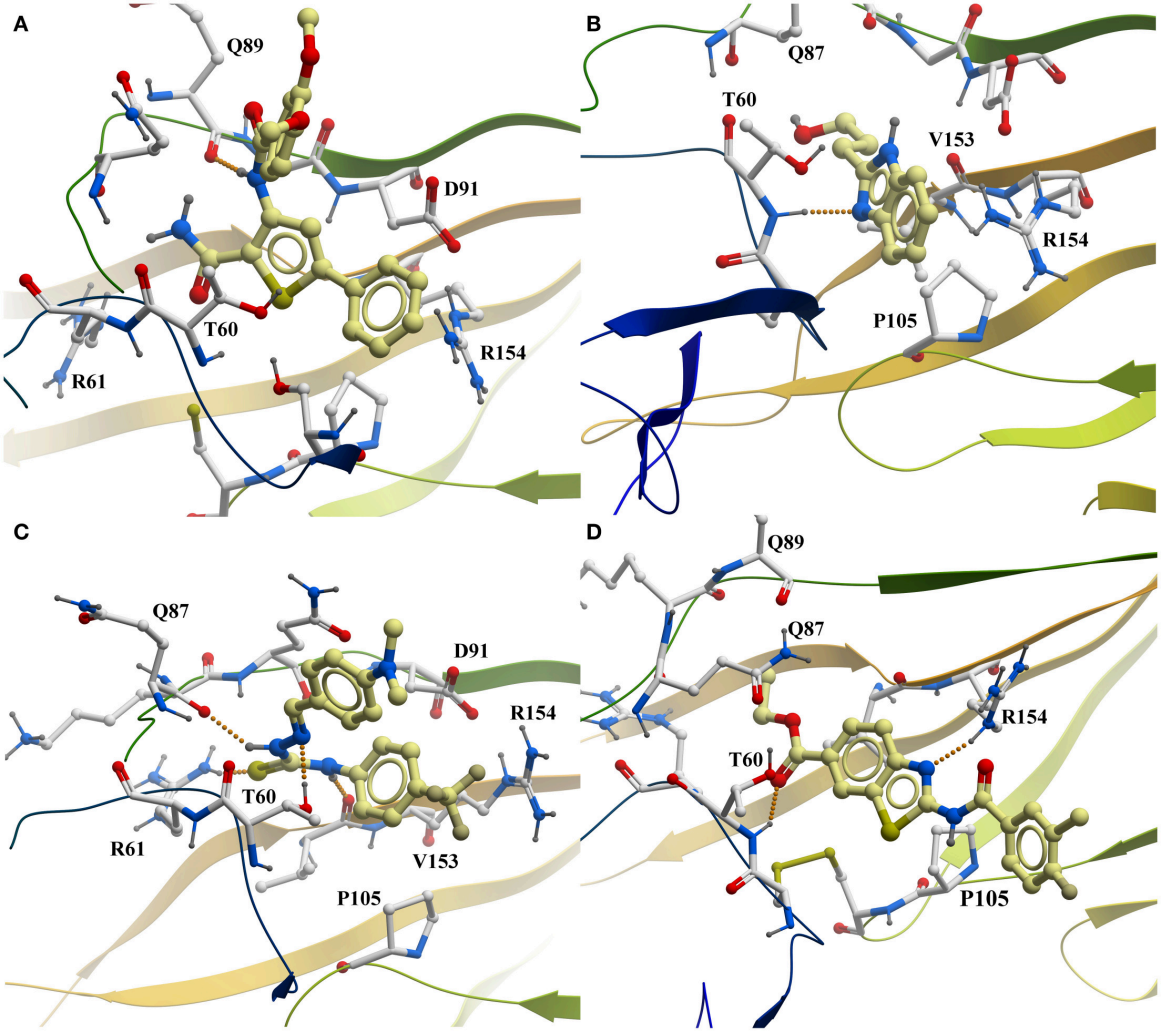
Predicted interaction of active compounds PTC12 **(A)**, BI03 **(B)**, **11 (C)**, and **8 (D)** within the E2 protein binding site, extracted from the molecular dynamics simulations. For simplicity, only aminoacids within 4 Å of the ligand and polar hydrogens are shown. Hydrogen bonds are shown as a line of orange colored spheres. Color code: ligand carbons, yellow; E2 protein carbons, white; oxygens, red; nitrogens, blue; sulfur, dark yellow; polar hydrogens, dark gray. Figure prepared with ICM (MolSoft LLC, La Jolla, CA).

**Figure 4:**
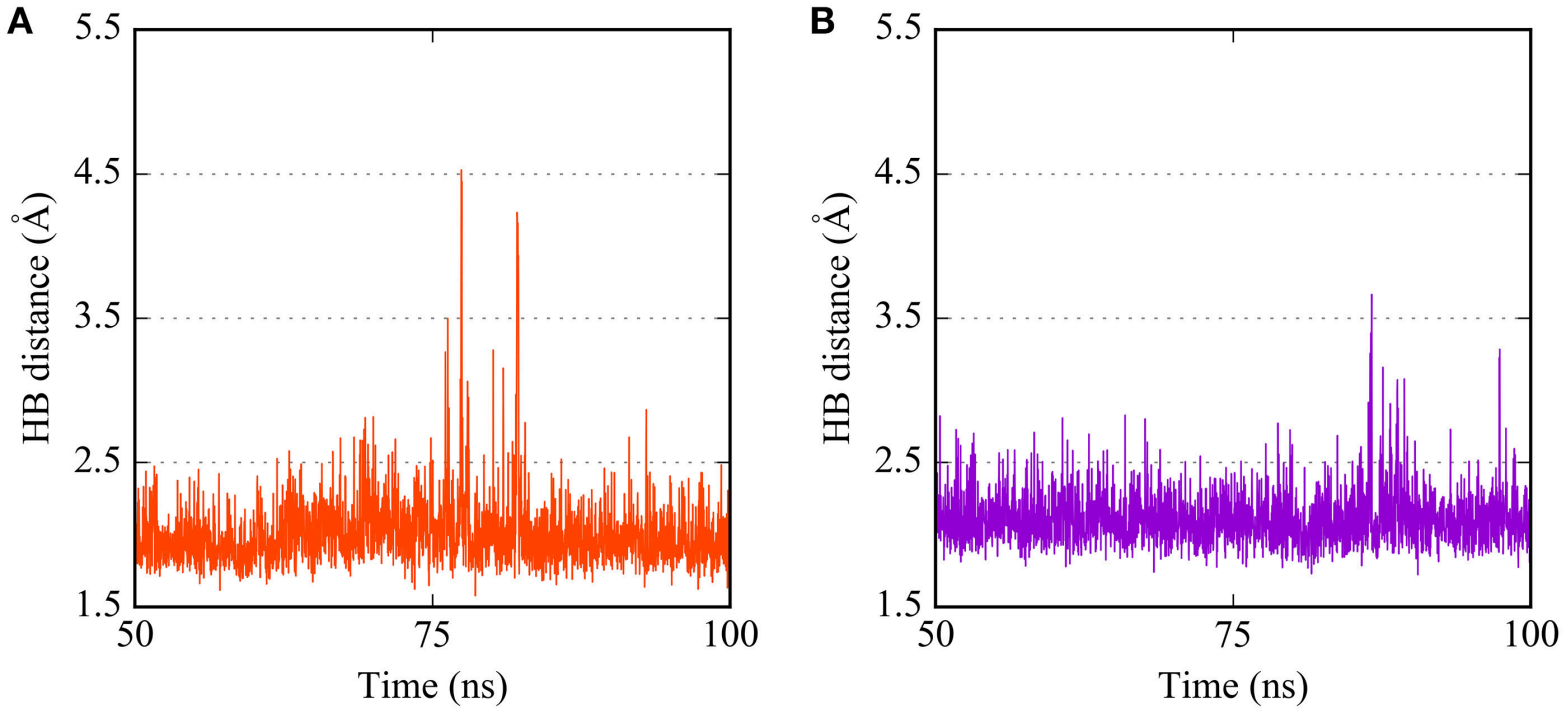
Time dependence of hydrogen bond (HB) distances between **(A)** the carbonyl O of Gln89 and the benzamide H of PTC12 ligand, and **(B)** the backbone amide H of Thr60 and the N2 atom of BI03 ligand.

The predicted binding mode of **PTC12** within the E2 protein is shown in Figures 2, 3. The 3,4-dimethoxybenzamide group remained exposed to the solvent, whereas the thiophene ring made contacts with Asp91, Thr60, and Arg61. The system also presented a strong hydrogen bond between the benzamide group of the ligand and the carbonyl O of Gln89, exhibiting an interatomic distance of ~2 Å and an angle of 160° during the last 50 ns simulation (Figure 4). A moderate hydrogen bond between the NH of the thiophencarboxamide and the carbonyl O Gln89 was detected, with interatomic distances and angles closer to 2.5 Å and 140°, respectively. This group was also intermittently exposed to the solvent through a narrow channel. A stable cation-π interaction between the aromatic ring of the 3, 4 dimethoxyphenyl group and Arg154 was observed throughout the simulation, with a N^+^-ring centroid distance below 6 Å at all times and a favorable θ angle below 40° during half of the last 50 ns simulation (Marshall et al., 2009).

The predicted interaction of **BI03** is shown in Figure 3. This ligand also presented a strong hydrogen bond between N2 and the backbone amide H of Thr60, with an average interatomic distance of 2.1 Å (Figure 4) and an angle of ~165° during the final half of the simulation. A stable cation-π interaction was also found in this case between the ligand ring and Arg154, showing again distances and θ angles below 6 Å and 40°, respectively for most of the final 50 ns of the simulation. A moderate hydrogen bond was also formed between the ring and the HO atom of Thr60. The system was further stabilized by close contacts with Thr60, Gln87, Arg154, Val153, and Pro105, while the ligand ring, NH and OH groups were mainly exposed to the solvent.

Compound **11** is shown within its predicted binding site in Figure 3 Two stable hydrogen bond occurred between the HN atoms of the ligand and the carbonyl O atom in Val153 and Gln87. In both cases interatomic distances and angles were very favorable with average values of 2 Å and 155° respectively. The cation-π interaction between Arg154 and the aromatic ring of 4-trifluoromethylphenyl group was less favorable than for the other compounds showing higher N^+^-ring centroid distances and θ angles, probably due to a moderate interaction between the CF_3_ group and the charged portion of Arg154. The ligand made contacts with Asp91, Arg61, Arg154, Gln87, Val153, Pro105, and Thr60 while the CF_3_ and NMe_2_ groups were mostly exposed to the solvent.

The predicted binding mode of compound **8** is shown in Figure 3. This pose was selected as the most probable one based on the analysis of the interactions and binding free energy estimations. In the last half of the simulation, a moderate hydrogen bond between N1 (N with no H) and the side chain of Arg154 was observed, with average interactomic distances of 2.5 Å and angles of ~140°. No cation-π interaction was detected, and the charged portion of Arg154 seemed to interact strongly with the amide group of the ligand. The 3-chloro-4-fluorobenzamide remained exposed to the solvent and there were close contacts of the ligand with Ser57, Thr60, Gln87, Gln89, Pro105, and Arg154.

Overall, molecular dynamics simulations reveal a common pattern of interactions with the binding site in E2. Taken together with previous studies on the mode of action (Pascual et al., 2018), our data support binding of active compounds to E2. Further studies including *in vitro* binding to the recombinant protein are still required to confirm the interaction of active compounds with E2.

## Conclusions

We have undertaken a structure-based virtual screening approach to identify small-molecules that dock into the druggable binding site at the interface between domains I and II of the E2 of BVDV, a virus responsible of both acute and persistent infections in cattle, with the consequent financial losses to the livestock industry each year. Around a million compounds were screened, and after chemical clustering, the top nineteen lead candidates were selected, and either purchased or synthesized, and evaluated in a reporter-based assay for antiviral activity. Five of these compounds exhibited IC_50_ values in the low micromolar range. The likely binding determinants of these compounds is supported by molecular dynamics simulations, where a common pattern of interaction with the binding site in E2 could be identified. These findings should benefit the design of novel and improved BVDV antivirals.

## Author contributions

MB and CNC: Designed and supervised the study; NSA, MGA, and CNC: Performed the computational simulations; MB and CNC: The virtual screening; ESL, GAF, and MB: Conducted chemical synthesis; MJP and FM: Performed biological experiments under the supervision of DEA; MB, DEA, and CNC: Analyzed data. All authors were involved in the preparation of the manuscript and approved the final version.

### Conflict of interest statement

The authors declare that the research was conducted in the absence of any commercial or financial relationships that could be construed as a potential conflict of interest.

## Supplementary material

The Supplementary Material for this article including MD plots, compound synthesis data, tested compounds codes, and NMR spectra can be found online at: https://www.frontiersin.org/articles/10.3389/fchem.2018.00079/full#supplementary-material

## References

[B1] Abagyan R. Totrov M. Kuznetsov D. (1994). ICM - a new method for protein modeling and design - applications to docking and structure prediction from the distorted native conformation. J. Comput. Chem. 15, 488–506. 10.1002/jcc.540150503

[B2] Abraham M. J. Murtola T. Schulz R. Páll S. Smith J. C. Hess B. (2015). GROMACS: high performance molecular simulations through multi-level parallelism from laptops to supercomputers. SoftwareX 1, 19–25. 10.1016/j.softx.2015.06.001

[B3] Baell J. B. Holloway G. A. (2010). New substructure filters for removal of pan assay interference compounds (PAINS) from screening libraries and for their exclusion in bioassays. J. Med. Chem. 53, 2719–2740. 10.1021/jm901137j 20131845

[B4] Becher P. Tautz N. (2011). RNA recombination in pestiviruses: cellular RNA sequences in viral genomes highlight the role of host factors for viral persistence and lethal disease. RNA Biol. 8, 216–224. 10.4161/rna.8.2.14514 21358277

[B5] Berendsen H. J. C. Postma J. P. M. Gunsteren W. F. van DiNola A. Haak J. R. (1984). Molecular dynamics with coupling to an external bath. J. Chem. Phys. 81, 3684–3690. 10.1063/1.448118

[B6] Brand C. S. Hocker H. J. Gorfe A. A. Cavasotto C. N. Dessauer C. W. (2013). Isoform selectivity of adenylyl cyclase inhibitors: characterization of known and novel compounds. J. Pharmacol. Exp. Ther. 347, 265–275. 10.1124/jpet.113.208157 24006339 PMC3807061

[B7] Callens N. Brügger B. Bonnafous P. Drobecq H. Gerl M. J. Krey T.. (2016). Morphology and molecular composition of purified bovine viral diarrhea virus envelope. PLoS Pathog. 12:e1005476. 10.1371/journal.ppat.1005476 26939061 PMC4777508

[B8] Cavasotto C. N. Orry A. J. W. (2007). Ligand docking and structure-based virtual screening in drug discovery. Curr. Top. Med. Chem. 7, 1006–1014. 10.2174/156802607780906753 17508934

[B9] Cavasotto C. N. Ortiz M. A. Abagyan R. A. Piedrafita F. J. (2006). *In silico* identification of novel EGFR inhibitors with antiproliferative activity against cancer cells. Bioorg. Med. Chem. Lett. 16, 1969–1974. 10.1016/j.bmcl.2005.12.067 16413185

[B10] Collett M. S. Larson R. Belzer S. K. Retzelt E. (1988). Proteins encoded by bovine viral diarrhea virus : the genomic organization of a pestivirus. Virology 165, 200–208. 10.1016/0042-6822(88)90673-3 2838958

[B11] El Omari K. Iourin O. Harlos K. Grimes J. M. Stuart D. I. (2013). Structure of a pestivirus envelope glycoprotein E2 clarifies its role in cell entry. Cell Rep. 3, 30–35. 10.1016/j.celrep.2012.12.001 23273918 PMC3607223

[B12] Fulton R. W. Ridpath J. F. Confer A. W. Saliki J. T. Burge L. J. Payton M. E. (2003). Bovine viral diarrhoea virus antigenic diversity: impact on disease and vaccination programmes. Biologicals 31, 89–95. 10.1016/S1045-1056(03)00021-6 12770537

[B13] He W. Elizondo-Riojas M. A. Li X. Lokesh G. L. Somasunderam A. Thiviyanathan V.. (2012). X-aptamers: a bead-based selection method for random incorporation of druglike moieties onto next-generation aptamers for enhanced binding. Biochemistry 51, 8321–8323. 10.1021/bi300471d 23057694 PMC3924539

[B14] Hornak V. Abel R. Okur A. Strockbine B. Roitberg A. Simmerling C. (2006). Comparison of multiple Amber force fields and development of improved protein backbone parameters. Proteins 65, 712–725. 10.1002/prot.21123 16981200 PMC4805110

[B15] Irwin J. J. Shoichet B. K. (2005). ZINC–a free database of commercially available compounds for virtual screening. J. Chem. Inf. Model. 45, 177–182. 10.1021/ci049714+ 15667143 PMC1360656

[B16] Jorgensen W. L. (2009). Efficient drug lead discovery and optimization. Acc. Chem. Res. 42, 724–733. 10.1021/ar800236t 19317443 PMC2727934

[B17] Jorgensen W. L. (2005). QikProp, v 3.0. New York, NY: Schrödinger, LLC.

[B18] Kampmann T. Yennamalli R. Campbell P. Stoermer M. J. Fairlie D. P. Kobe B.. (2009). *In silico* screening of small molecule libraries using the dengue virus envelope E protein has identified compounds with antiviral activity against multiple flaviviruses. Antiviral Res. 84, 234–241. 10.1016/j.antiviral.2009.09.007 19781577

[B19] King F. E. Acheson R. M. Spensley P. C. (1948). 275. Benziminazole analogues of paludrine. J. Chem. Soc. 1948, 1366–1371. 10.1039/jr9480001366 18893616

[B20] Lagorce D. Sperandio O. Baell J. B. Miteva M. A. Villoutreix B. O. (2015). FAF-Drugs3: a web server for compound property calculation and chemical library design. Nucleic Acids Res. 43, W200–W207. 10.1093/nar/gkv353 25883137 PMC4489254

[B21] Lanyon S. R. Hill F. I. Reichel M. P. Brownlie J. (2014). Bovine viral diarrhoea : pathogenesis and diagnosis. Vet. J. 199, 201–209. 10.1016/j.tvjl.2013.07.024 24053990

[B22] Leal E. S. Aucar M. G. Gebhard L. G. Iglesias N. G. Pascual M. J. Casal J. J.. (2017). Discovery of novel dengue virus entry inhibitors via a structure-based approach. Bioorg. Med. Chem. Lett. 27, 3851–3855. 10.1016/j.bmcl.2017.06.049 28668194

[B23] Li Y. Wang J. Kanai R. Modis Y. (2013). Crystal structure of glycoprotein E2 from bovine viral diarrhea virus. Proc. Natl. Acad. Sci. U. S. A. 110, 6805–6810. 10.1073/pnas.1300524110 23569276 PMC3637714

[B24] Lipinski C. A. Lombardo F. Dominy B. W. Feeney P. J. (1997). Experimental and computational approaches to estimate solubility and permeability in drug discovery and development settings. Adv. Drug Deliv. Rev. 46, 3–26. 10.1016/S0169-409X(96)00423-1 11259830

[B25] Liu K. Kokubo H. (2017). Exploring the stability of ligand binding modes to proteins by molecular dynamics simulations: a cross-docking study. J. Chem. Inf. Model. 57, 2514–2522. 10.1021/acs.jcim.7b00412 28902511

[B26] Marshall M. S. Steele R. P. Thanthiriwatte K. S. Sherrill C. D. (2009). Potential energy curves for cation-π interactions: off-axis configurations are also attractive. J. Phys. Chem. A 113, 13628–13632. 10.1021/jp906086x 19886621

[B27] Nemethy G. Gibson K. D. Palmer K. A. Yoon C. N. Paterlini M. G. Zagari A. (1992). Energy parameters in polypeptides. 10. Improved geometrical parameters and nonbonded interactions for use in the ECEPP/3 algorithm, with application to proline-containing peptides. J. Phys. Chem. 96, 6472–6484. 10.1021/j100194a068

[B28] Newcomer B. W. Chamorro M. F. Walz P. H. (2017). Vaccination of cattle against bovine viral diarrhea virus. Vet. Microbiol. 206, 78–83. 10.1016/j.vetmic.2017.04.003 28400145

[B29] Newcomer B. W. Givens M. D. (2013). Approved and experimental countermeasures against pestiviral diseases : bovine viral diarrhea, classical swine fever and border disease. Antiviral Res. 100, 133–150. 10.1016/j.antiviral.2013.07.015 23928259

[B30] Newcomer B. W. Marley M. S. Galik P. K. Walz P. H. Zhang Y. Riddell K. P.. (2012). Antiviral treatment of calves persistently infected with bovine viral diarrhoea virus. Antivirus Chem. Chemother. 22, 171–179. 10.3851/IMP1903 22182713

[B31] Pascual M. J. Merwaiss F. Leal E. Quintana M. E. Capozzo A. V. Cavasotto C. N.. (2018). Structure-based drug design for envelope protein E2 uncovers a new class of bovine viral diarrhea inhibitors that block virus entry. Antiviral Res. 149, 179–190. 10.1016/j.antiviral.2017.10.010 29031833

[B32] Ridpath J. F. (2003). BVDV genotypes and biotypes: practical implications for diagnosis and control. Biologicals 31, 127–131. 10.1016/S1045-1056(03)00028-9 12770544

[B33] Ronecker S. Zimmer G. Herrler G. Greiser-wilke I. Grummer B. (2008). Formation of bovine viral diarrhea virus E1 – E2 heterodimers is essential for virus entry and depends on charged residues in the transmembrane domains. J. Gen. Virol. 89, 2114–2121. 10.1099/vir.0.2008/001792-0 18753220

[B34] Spyrakis F. Cavasotto C. N. (2015). Open challenges in structure-based virtual screening: receptor modeling, target flexibility consideration and active site water molecules description. Arch. Biochem. Biophys. 583, 105–119. 10.1016/j.abb.2015.08.002 26271444

[B35] Totrov M. Abagyan R. Raffa R. B. (2001). Protein-ligand docking as an energy optimization problem, in Drug-receptor Thermodynamics: Introduction and Experimental Applications, ed Raffa R. B. (New York, NY: John Wiley and Sons), 603–624.

[B36] Wang Q. Y. Patel S. J. Vangrevelinghe E. Hao Y. X. Rao R. Jaber D.. (2009). A small-molecule dengue virus entry inhibitor. Antimicrob. Agents Chemother. 53, 1823–1831. 10.1128/AAC.01148-08 19223625 PMC2681551

[B37] Zhou Z. Khaliq M. Suk J. E. Patkar C. Li L. Kuhn R. J.. (2008). Antiviral compounds discovered by virtual screening of small-molecule libraries against dengue virus E protein. ACS Chem. Biol. 3, 765–775. 10.1021/cb800176t 19053243 PMC2782732

