# Supplementary material for: Discovery of Novel Bovine Viral Diarrhea Inhibitors Using Structure-Based Virtual Screening on the Envelope Protein E2
Source: Front Chem. 2018 Mar 26;6:79. doi: 10.3389/fchem.2018.00079 (PMC5879447; doi:10.3389/fchem.2018.00079)
Supplement: Supplementary file 1 [file DataSheet1.pdf]

Supplementary Material

Structure-based virtual screening and molecular dynamics simulations to identify novel bovine viral diarrhea envelope protein E2 inhibitors

Mariela Bollini[1,*] Emilse S. Leal[1], Natalia S. Adler[2], M. Gabriela Aucar[2], Gabriela A. Fernández[1], María J. Pascual[3], Fernando Merwaiss[3], Diego E. Alvarez[3], Claudio N. Cavasotto[2,*]

*** Correspondence:** mariela.bollini@cibion.conicet.gov.ar; cnc@cavasotto-lab.net ccavasotto@ibioba-mpsp-conicet.gov.ar

Table of content page

Table of content	page
Molecular dynamics RMSD plots	2
Synthesis and HRMS data of compounds **5**, **12**, **13**, **17**	2-4
Table of tested compounds codes	4
^{1}H-NMR and ^{13}C-NMR spectra	5-10
References	9-10

1 Supplementary Data

1.1 Computational Chemistry

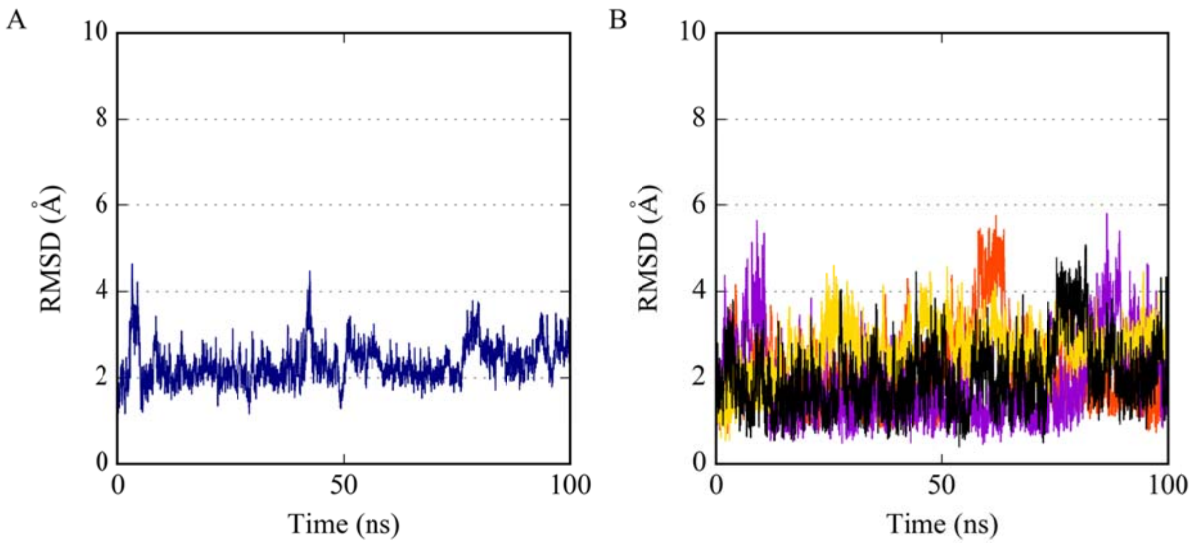

Fig S1. RMSD as a function of time for (A) protein backbone atoms, and (B) the ligands PTC12 (orange), BI03 (violet), 11 (yellow) and 8 (black).

2.1 Synthetic procedure of compounds 5, 12, 13 and 17 from the ZINC library

2.1.1 Compound 5

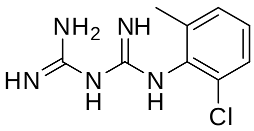

The compound was synthesized according to literature **5** (Kothayer et al. 2014)**.** Yield 72 %. HR-MS (ES) calcd for $C_9H_{12}ClN_5$ $[M+H]^+$ 226.0781found 226.0801.

2.1.2 Compound 12

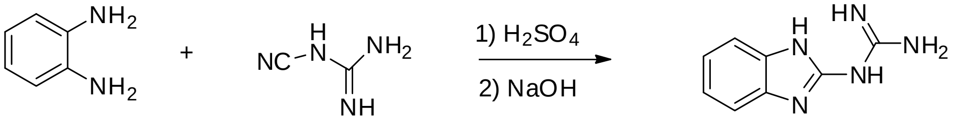

12

Scheme S1

The compound was synthesized according to literature (King, Acheson, and Spensley 1948) with modifications. Briefly, ortho phenylendiamine (0.5g, 5 mmol) was dissolved in 10 % H_2SO_4 (5 mL) and dicyandiamide (0.58 g, 6 mmol) was added. The reaction mixture was refluxed overnight. After this time, NaOH 50 % (1 mL) was added and heated for 2 h. The reaction mixture was cooled, and the solid was collected by filtration, washed with water, dried and purified by silica gel column chromatography eluting with DCM/MeOH (98:2 to 90:10). To yield a white solid (0.4 g, 50 %) HR-MS (ES) *m/z* calcd for $C_8H_9N_5$ $[M+H]^+$ 176.0936 found 176.0938 $C_8H_9N_5Na$ $[M+Na]^+$ 198.0756 found 198.0757.

2.1.3 Compound 13

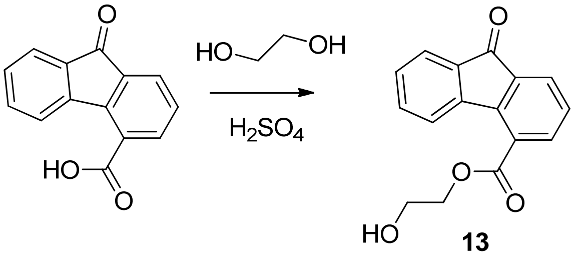

Scheme S2.

An excess of ethylene glycol (20 ml) was added to 9-fluorenone-4-carboxylic acid (0.2 g, 0.9 mmol) and catalytic amount of H_2SO4.The mixture was stirred at room temperature for 12 h, then the solution was evaporated under vacuum. The residue was dissolved in CH_2Cl_2 and washed with a saturated solution of $NaHCO_3$ and then with distilled water. The organic layer was dried over anhydrous Na_2SO_4 and evaporated under vacuum. The product was purified by silica gel chromatography (CH_2Cl_2/CH_3OH 9:1) to yield a white solid (0.18 g, 78 %) HR-MS (ES) *m/z* calcd $C_{16}H_{12}O_4$ $[M+H]^+$ 269.0736 found 269.0741

2-hydroxyethyl 9-oxo-9H-fluorene-4-carboxylate

2.1.4 Compound 17

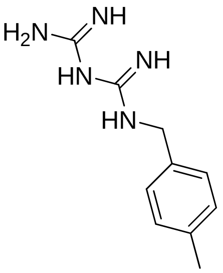

The compound was synthesized according to literature (Magri et al. 2015) Yield (61 %). HR-MS (ES) calcd for $C_{10}H_{15}N_5$ $[M+H]^+$ 206.1327 found 206.1312

3. Table of tested compounds codes

Compounds	Library/code
**1**	**ZINC26579141**

**2**	**ZINC35446539**
**3**	**ZINC130163**
**4**	**Maybridge, RJF00547**
**5**	**ZINC15924305**
**6**	**ZINC1040053**
**7**	**ZINC4620907**
**8**	**ZINC1032412**
**9**	**ZINC13464017**
**10**	**ZINC261907**
**11**	**In house library**
**12**	**Maybridge JFD01760**
**13**	**Maybridge BTB13867**
**14**	**In house library**
**15**	**In house library**
**16**	**In house library**
**17**	**ZINC390716**
**BI03**	**Maybridge BTB05684**
**PICT12**	**Maybridge GK03725**

4. Supplementary Figures ^{1}H-NMR and ^{13}C-NMR spectra

4.1 Compound 11

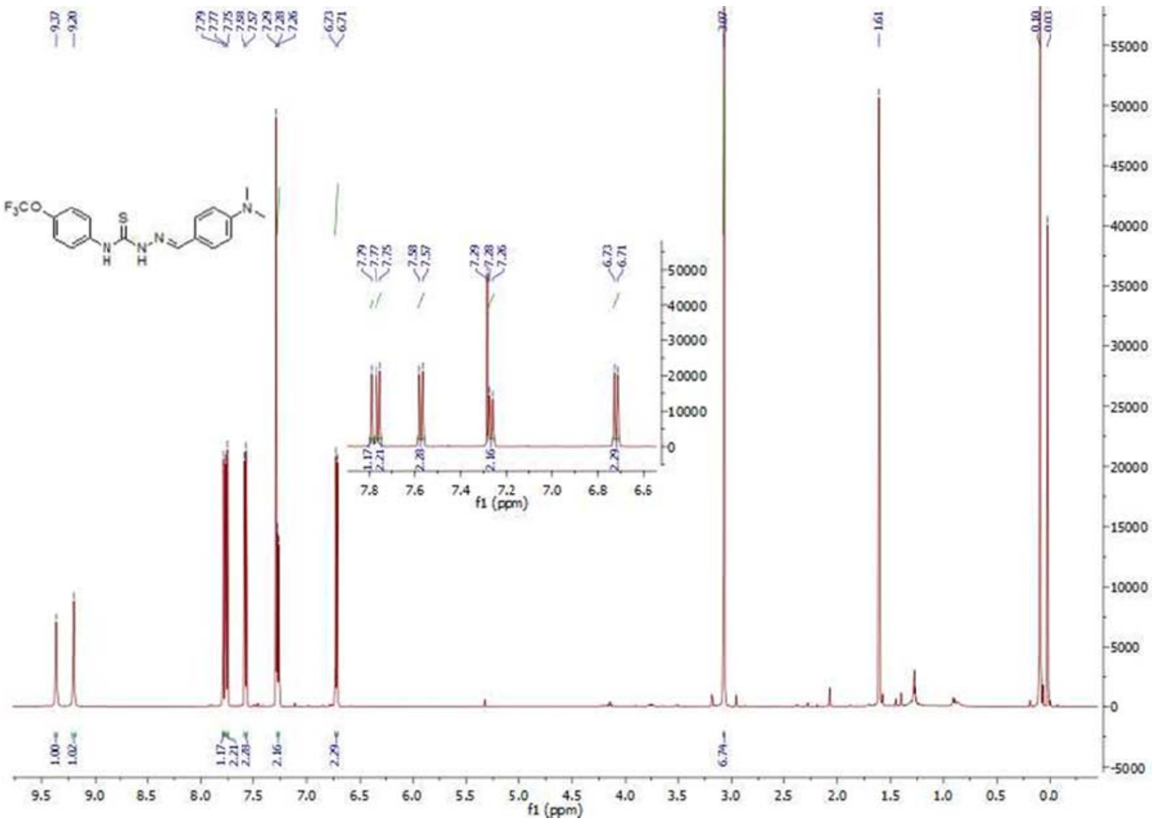
F3CO
f1 (ppm)

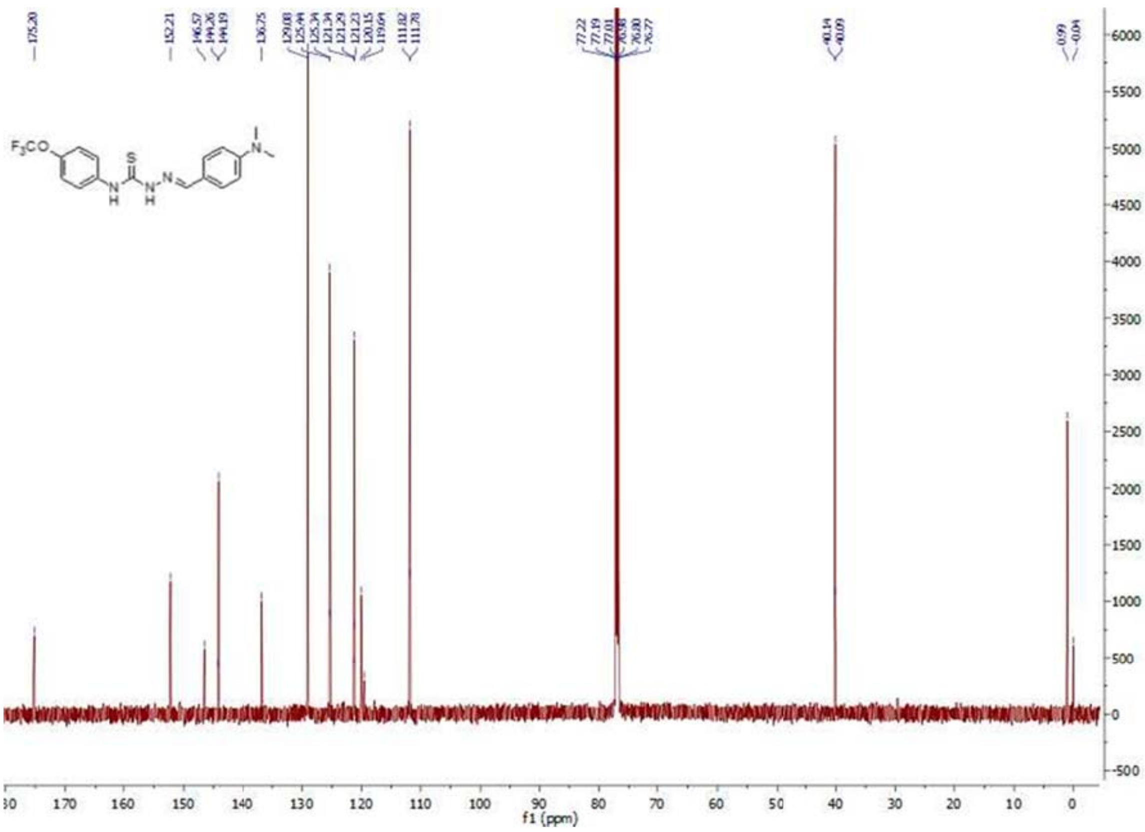
F3CO
f1 (ppm)

4.2 Compound 14

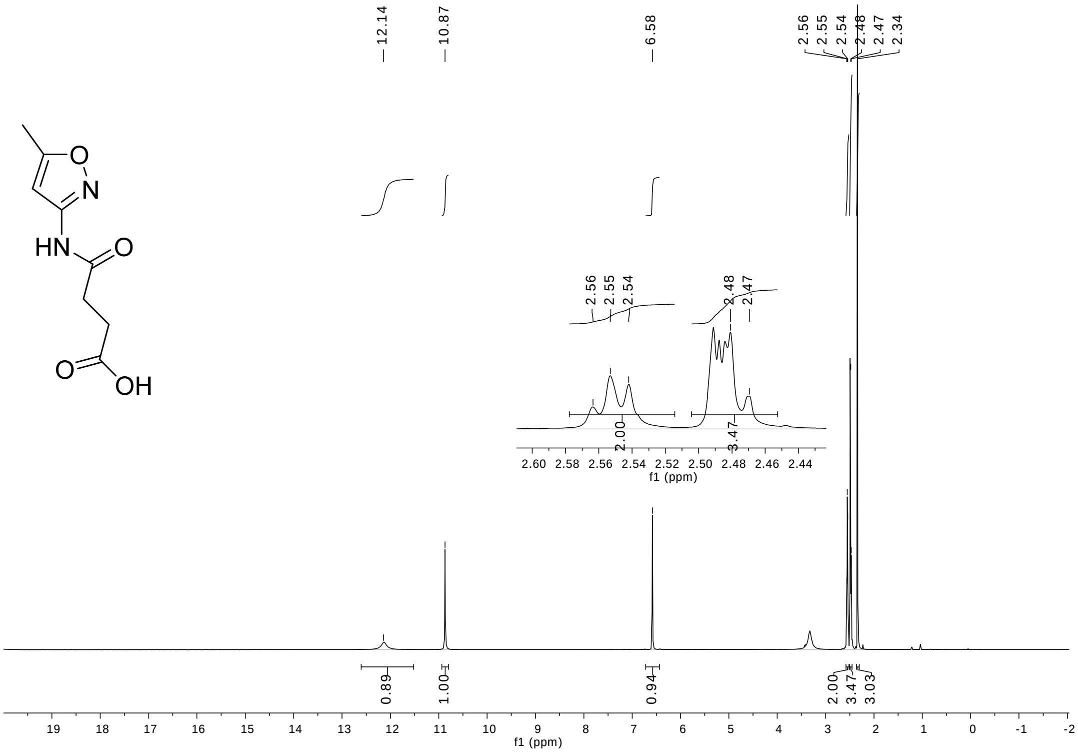

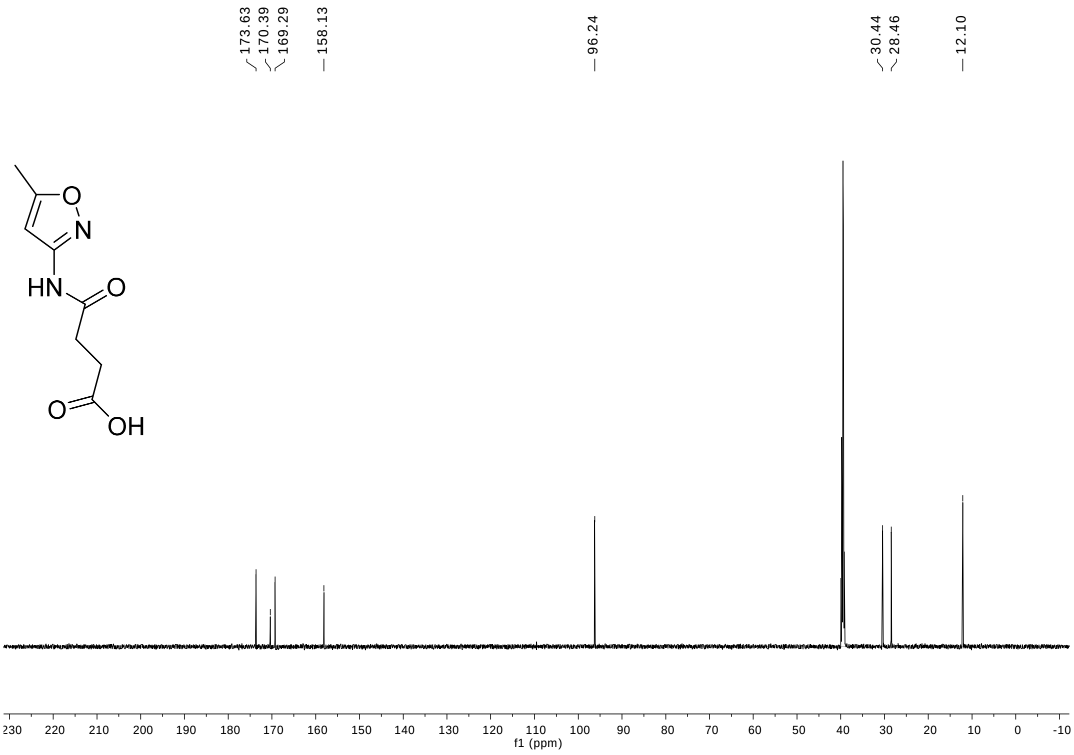

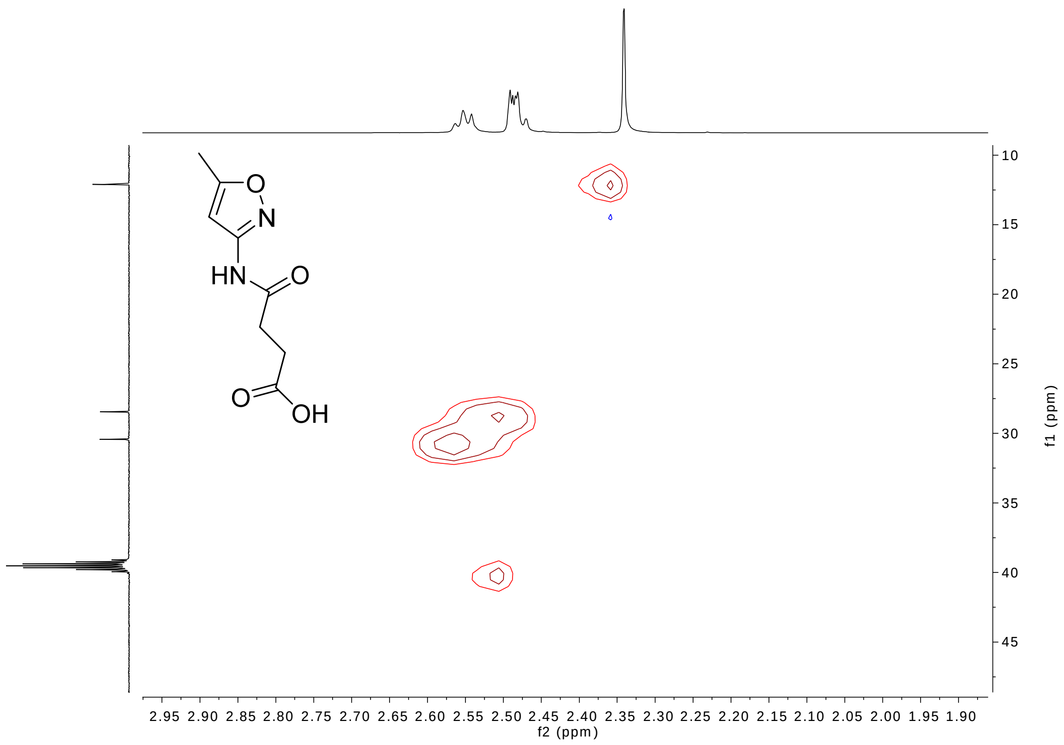

4.3 Compound 15

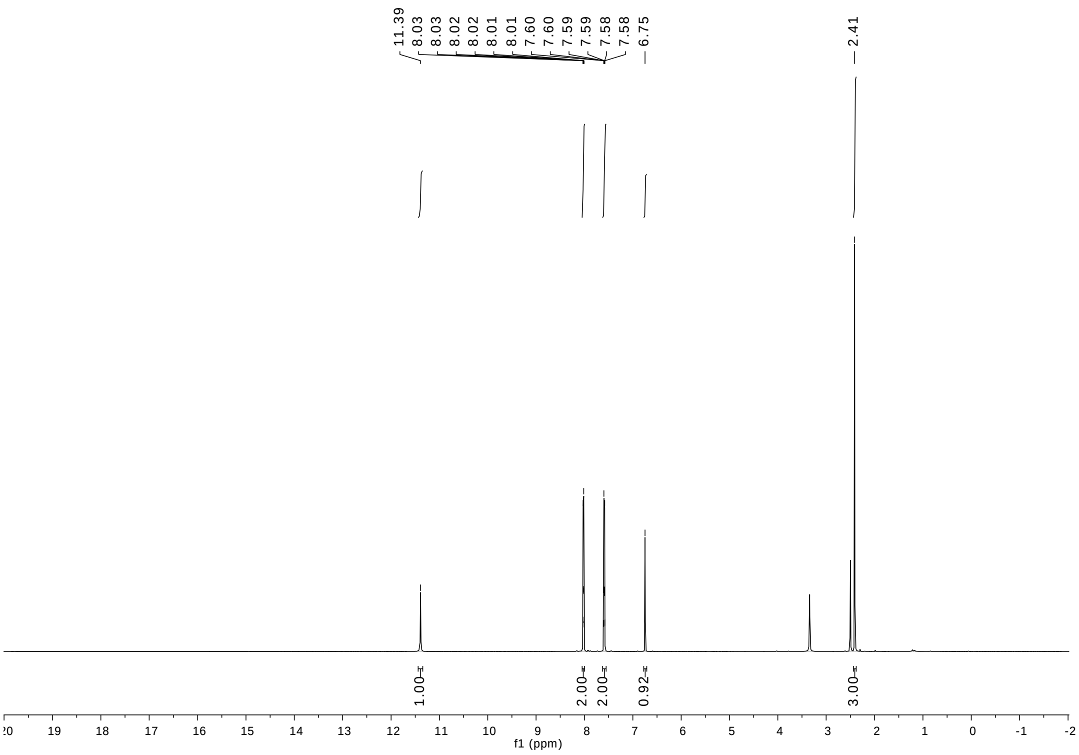

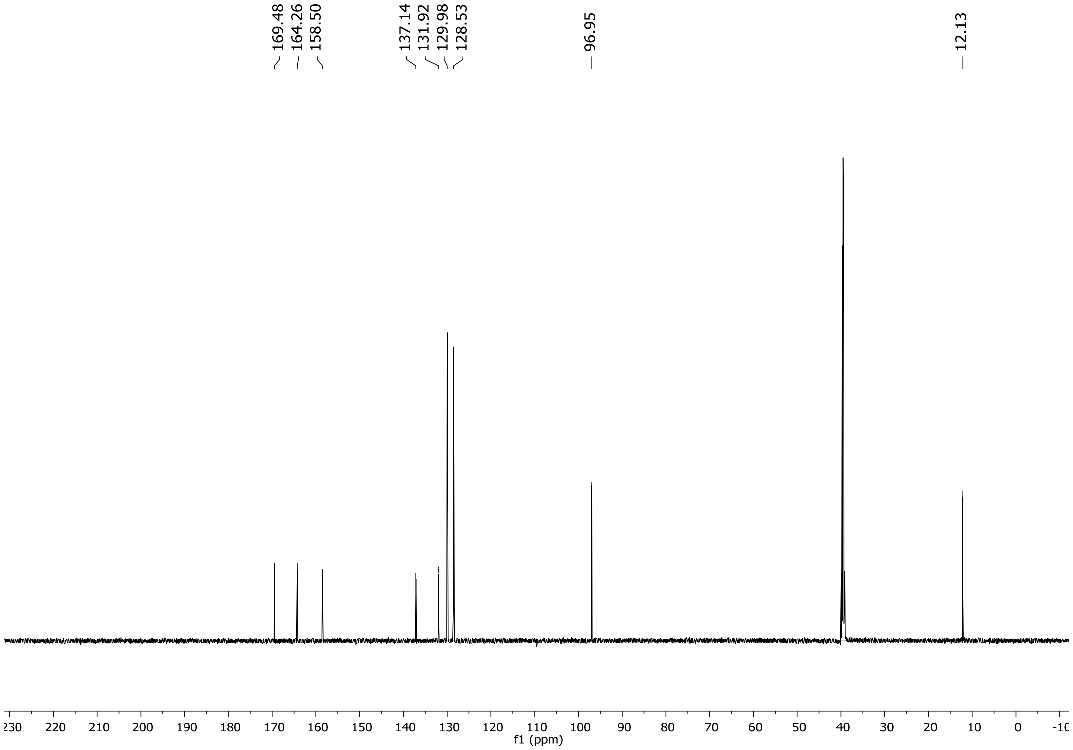

5. References

King, F E, R M Acheson, and P C Spensley. 1948. “275. Benziminazole Analogues of Paludrine.” *Journal of the Chemical Society (Resumed)*, no. 0. The Royal Society of Chemistry: 1366–71. doi:10.1039/JR9480001366.

Kothayer, Hend, Matteo Morelli, Ghali Brahemi, Abdalla A Elshanawani, Mansour E Abu Kull, Osama I El-Sabbagh, Malathy P V Shekhar, and Andrew D Westwell. 2014. “Optimised Synthesis of Diamino-Triazinylmethyl Benzoates as Inhibitors of Rad6B Ubiquitin Conjugating Enzyme.” *Tetrahedron Letters* 55 (51): 7015–18. doi:https://doi.org/10.1016/j.tetlet.2014.10.122.

Magri, Andrea, Roisin Reilly, Nicolò Scalacci, Marco Radi, Michael Hunter, Manon Ripoll, Arvind H Patel, and Daniele Castagnolo. 2015. “Rethinking the Old Antiviral Drug Moroxydine: Discovery of Novel Analogues as Anti-Hepatitis C Virus (HCV) Agents.” *Bioorganic & Medicinal Chemistry Letters* 25 (22): 5372–76. doi:https://doi.org/10.1016/j.bmcl.2015.09.029.
